# Impact of Aberrant β-Catenin Pathway on Cholangiocarcinoma Heterogeneity

**DOI:** 10.3390/cells12081141

**Published:** 2023-04-12

**Authors:** Elisa Lozano, Paula Sanchon-Sanchez, Ana Morente-Carrasco, Luis Miguel Chinchilla-Tábora, José L. Mauriz, Paula Fernández-Palanca, Jose J. G. Marin, Rocio I. R. Macias

**Affiliations:** 1 Experimental Hepatology and Drug Targeting (HEVEPHARM) Group, Biomedical Research Institute of Salamanca (IBSAL), University of Salamanca, 37007 Salamanca, Spain; 2 Center for the Study of Liver and Gastrointestinal Diseases (CIBERehd), Carlos III National Institute of Health, 28029 Madrid, Spain; 3 Area of Physiology, Faculty of Health Sciences, University Rey Juan Carlos, 28032 Alcorcón, Madrid, Spain; 4 Pathology Service, University Hospital and IBSAL, 37007 Salamanca, Spain; 5 Institute of Biomedicine (IBIOMED), Universidad de León, 24071 León, Spain

**Keywords:** biliary cancer, β-catenin, CCA, personalized treatment, pharmacogenomic, Wnt

## Abstract

The poor prognosis of most cases of advanced cholangiocarcinoma (CCA) constitutes a severe problem in modern oncology, which is aggravated by the fact that the incidence of this liver cancer is increasing worldwide and is often diagnosed late, when surgical removal is not feasible. The difficulty of dealing with this deadly tumor is augmented by the heterogeneity of CCA subtypes and the complexity of mechanisms involved in enhanced proliferation, apoptosis avoidance, chemoresistance, invasiveness, and metastasis that characterize CCA. Among the regulatory processes implicated in developing these malignant traits, the Wnt/β-catenin pathway plays a pivotal role. Alteration of β-catenin expression and subcellular localization has been associated with worse outcomes in some CCA subtypes. This heterogeneity, which also affects cellular and in vivo models commonly used to study CCA biology and anticancer drug development, must be taken into account for CCA investigation to more accurately extrapolate basic laboratory research to the clinical situation. A better understanding of the altered Wnt/β-catenin pathway in relationship with the heterogeneous forms of CCA is mandatory for developing novel diagnostic tools and therapeutic strategies for patients suffering from this lethal disease.

## 1. Introduction

Cholangiocarcinoma (CCA) is an extremely heterogeneous malignancy arising in cholangiocytes, i.e., the epithelial cells that line the bile ducts [1]. CCA constitutes the second most frequent primary hepatobiliary cancer after hepatocellular carcinoma (HCC). This cancer is characterized by a worse prognosis than HCC, with a median overall survival (OS) lower than 11 months in patients with advanced tumors [2] and a 5-year OS that is also low (5–10%) in these cases [3]. According to the primary anatomical origin, CCAs are classified as intrahepatic (iCCA) and extrahepatic (eCCA), with the latter being divided into perihilar (pCCA), and distal (dCCA), all of which are considered different entities from a biological and clinical point of view. Moreover, two types of iCCA are now recognized: the “large duct type”, which resembles pCCA or dCCA, and the “small duct type”. Additional histological subtypes included in small duct–type iCCAs, such as cholangiolo-carcinoma and iCCA with a ductal plaque malformation pattern, as well as combined hepato-cholangiocarcinoma (cHCC-CCA), further increase the complexity and heterogeneity of these tumors [4].

A recent meta-analysis revealed that bile duct cysts and stones, cirrhosis, and viral hepatitis B and C, but not hypertension and obesity, are risk factors for all types of CCA [5]; however, the largest pan-European observational study found that overweight/obesity is associated with iCCA, whereas primary sclerosing cholangitis is a major risk factor for pCCA, as choledocholithiasis is for dCCA [2]. In eastern countries where CCA is highly prevalent, such as in Thailand, infection with *Opisthorchis viverrini*, advanced age, and alcohol consumption have been described as risk factors for CCA development [6].

An important proportion of CCA cases arises in patients with non-diagnosed underlying diseases, but it is accepted that chronic cholestasis/inflammation leads to cholangiocarcinogenesis by inducing DNA damage, apoptosis evasion, promotion of cell proliferation, and neoangiogenesis [7]. Bile acid accumulation in liver does not directly induce carcinogenesis but may favor the development of CCA by playing a cocarcinogenic role by stimulating bile duct proliferation, enhancing inflammation, and reducing FXR-dependent chemoprotection [8]. Moreover, chronic inflammation results in accumulation of genetic and epigenetic alterations, which leads to aberration of oncogenes and tumor suppressors [9].

According to their transcriptomic profiles, iCCAs are divided into the “inflammation” and the “proliferation” subtypes [10]; the first subtype is characterized by activation of immune-related signaling pathways, and the second is associated with activation of oncogenic pathways and worse OS. Altered oncogenic pathways include tyrosine kinase receptor (TKR)-mediated signaling, RAS-RAF-ERK, PI3K-AKT-mTOR, insulin growth factor receptor 1, polo-like kinase 1, aurora kinase A, Wnt/β-catenin signaling pathways, and mutations in IDH1/IDH2 genes, among others [1,11], which lead to enhanced cell proliferation, migration, or invasion. These pathways are activated by the complex interaction of multiple extracellular ligands located in the tumor microenvironment, including proinflammatory cytokines, growth factors, bile acids, and others [12,13,14].

**Figure 1:**
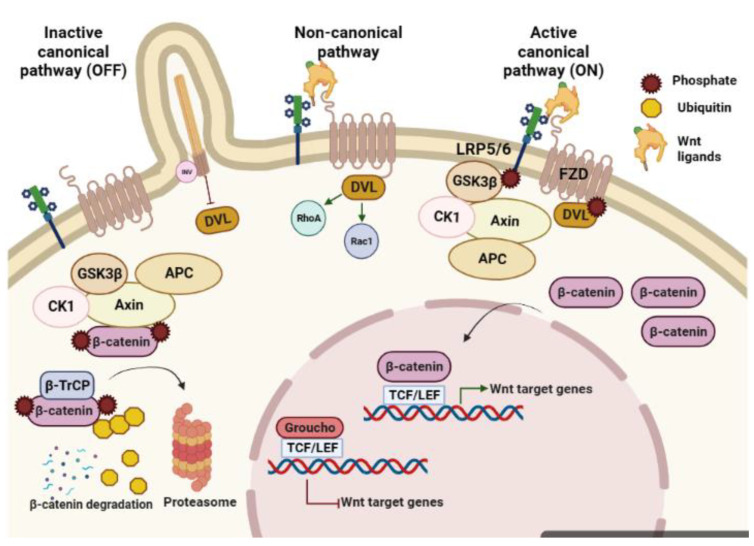
Schematic representation of canonical and non-canonical Wnt signaling pathways in cholangiocytes and the role of the primary cilia in modulating these pathways. In the absence of Wnt ligands (inactive, canonical Wnt/β-catenin pathway OFF), β-catenin is degraded by the destruction complex, and Wnt target genes are silenced. In the presence of Wnt ligands (active, Wnt/β-catenin pathway ON), β-catenin accumulates in the cytoplasm and translocates to the nucleus, promoting the expression of Wnt target genes. Furthermore, in the presence of Wnt ligands, the non-canonical pathway stimulates planar cell polarity through activation of small GTPases Rho and Rac, which induce cytoskeletal rearrangements. APC, adenomatous polyposis coli; CK1, casein kinase 1; DVL, Disheveled; FZD, frizzled receptor; GSK3β, glycogen synthase kinase 3 beta; INV, inversin; LRP5/6, low density lipoprotein receptor-related proteins 5/6; TCF/LEF, T-cell factor/lymphoid enhancer factor; β-TrCP, β-transducin repeats-containing protein. Created with BioRender.com (Accessed on 7 April 2023).

The Wnt/β-catenin signaling pathway is not the most important oncogenic pathway in CCA, but it certainly plays a role and contributes to the heterogeneity of this tumor. β-catenin is a component of adherens junctions, cell–cell adhesion complexes essential for adhesion and anchoring of the actin cytoskeleton [15], localized in the plasma membrane due to its association to cadherins; it is also a cytoplasmic transcription effector of the Wnt-signaling pathway. When this pathway is activated, β-catenin is translocated to the nucleus, where it regulates the expression of target genes (Figure 1). When there are no extracellular Wnt ligands, the canonical Wnt-β-catenin pathway is in an inactive state (Wnt-OFF), and β-catenin is attached to the removal complex formed by four proteins: Axin, adenomatous polyposis coli (APC) protein, glycogen synthase kinase 3β (GSK3β), and isoform-α of casein kinase 1 (CK1α) [16]. In this complex, β-catenin undergoes phosphorylation that allows its ubiquitination and degradation by the proteasome. In this situation, there is no transcription of Wnt target genes because nuclear transcription factors are linked to inhibitors such as Groucho. In the presence of Wnt ligands, Wnt signaling is in an active state (Wnt-ON). Wnt ligands bind to the Frizzled membrane receptor (FZD), favoring the formation of a complex with low-density lipoprotein receptor-related proteins 5/6 (LRP5/6), which undergoes phosphorylation and binds Axin to the cytoplasmic end of LRP6, while Disheveled (DVL) binds to the C-terminal end of FZD. Axin sequestration avoids β-catenin phosphorylation and its degradation, so it accumulates in the cytoplasm and can be translocated to the nucleus, where β-catenin binds to several co-activators and to transcription factors, such as T-cell nuclear DNA-binding factor/lymphoid enhancer-binding factor (TCF/LEF), hypoxia-inducible factor 1α (HIF1α), forkhead box protein O (FOXO), and members of the sex-determining region Y box (SOX) family [17,18,19,20].

Wnt ligands are cysteine-rich glycoproteins of 350–400 amino acids (including Wnt2, Wnt3, Wnt5, Wnt7, and Wnt10) secreted by different cell types that can act in an autocrine or paracrine manner [21].

Wnt ligands can also trigger a β-catenin–independent signaling pathway, the non-canonical planar cell polarity (PCP) Wnt pathway, through activation of the small GTPases Rho and Rac, which induce cytoskeletal rearrangements and polarization of epithelial cells [22,23,24]. Loss or malformation of primary cilia has been associated with several cholangiopathies, such as polycystic kidney disease (PKD) or biliary atresia [25,26,27,28,29,30], and also with the development of CCA [31]. It has been demonstrated that cilia on cholangiocytes regulate the Wnt/β-catenin signaling pathway through the detection of extracellular signals and subsequent activation of downstream signaling pathways [32]. The inversin (INV) protein, localized in the basal body of primary cilia, interacts with and inhibits cytoplasmic DVL, thus acting as a molecular switch between the canonical and non-canonical Wnt pathways [33,34]. In the presence of primary cilia, INV mediates, and a portion of DVL is degraded by the proteasome, causing degradation of part of the β-catenin. Loss of primary cilia has been associated with higher levels of cytoplasmic DVL, which causes an increase of cytoplasmic and nuclear β-catenin levels and overexpression of its target genes.

Morphologically, CCA is characterized by an abundant desmoplastic stroma surrounding the tumor, with dense connective tissue, disorganized blood vessels, and inflammatory macrophages, which are considered an important source of Wnt ligands in these tumors. Activation of the Wnt/β-catenin signaling pathway is frequent in CCA and is associated with tumor cell proliferation [35]. Accordingly, blocking this pathway may have therapeutic potential. Thus, although several strategies have been investigated in this regard, much research in needed to confirm the usefulness of different options and to determine which patients might benefit from them.

## 2. Molecular Alterations in the Wnt/β-Catenin Pathway in CCA

As mentioned, it has been described that in CCA (similar to other tumors), Wnt/β-catenin activity is pathologically activated [35]. Mutations in downstream components of the Wnt signaling pathway have been identified in several types of human cancers, including gastrointestinal cancers, such as HCC or colon adenocarcinoma [36,37]. However, the components of the Wnt/β-catenin pathway are not frequently mutated in CCA.

### 2.1. Molecular Alterations in β-Catenin

In other liver tumors, Wnt signaling is aberrantly activated by the presence of gain-of-function mutations in β-catenin (*CTNNB1* gene), which increase protein stability. This type of mutation has been found in HCC with a frequency of ≈19% [38]. Moreover, in the pediatric liver cancer hepatoblastoma, *CTNNB1* is the most recurrently mutated gene, with a frequency of 50–90%, which leads to consider *CTNNB1* as a driving proto-oncogene [39,40,41]. However, *CTNNB1* mutations are usually absent in CCA. Some studies have not found *CTNNB1* mutations in CCA specimens [42,43], whereas others have detected some with very low frequency [44]. According to the COSMIC and CBioPortal databases, the frequency of mutation in the *CTNNB1* gene in CCA is <2% (Table 1).

### 2.2. Molecular Alterations in APC

One of the Wnt pathway tumor suppressor genes with the most mutations in patients with CCA is *APC*. In a cohort of 22 CCA patients, genetic alterations as a loss of heterozygosity in *APC* were found in ≈40% of cases, which was related to early stages of the disease [45]. The COSMIC and CBioPortal studies found the *APC* gene is mutated in about 3% of iCCA samples (Table 1). Other studies with analyses performed by next-generation sequencing (NGS) found a mutation frequency of ≈2% in the *APC* gene in patients with iCCA and gallbladder tumors [44,46]. NGS analysis of 17 tissue samples from patients with iCCA (mass-forming type) and 24 bile samples from patients with pCCA or dCCA (diffusely infiltrating type) identified *APC* mutations only in mass-forming iCCAs, namely one deletion and one single nucleotide polymorphism (SNP), with a frequency of 14.3% of cases [47]. Epigenetic alterations in this gene have also been described. The study by Yang et al. [48] including 72 patients with CCA, half iCCA and half eCCA, found that the APC promoter was methylated in 46% of patients with CCA. The frequency of methylation in this tumor suppressor gene was consistent, regardless of the anatomic location of the tumors. Both the down-regulation and the presence of inactivating mutations in the *APC* gene lead to a less effective degradation complex, which causes β-catenin accumulation in the cytoplasm and nuclei, resulting in an aberrantly activated pathway.

### 2.3. Alterations in AXIN1 and AXIN2

The same effect results from mutations in the *AXIN1* gene that generate inactive proteins. *AXIN1* has a mutation frequency of 1–2% in patients with CCA, according to data from CBioPortal and COSMIC (Table 1). A frequency of mutations in this gene of 15% of cases has also been reported [49]. Hypermutation and unique mutational signatures have been described in iCCA samples from young printing press workers exposed to haloalkanes (n = 4). *AXIN1* mutations affecting the protein (S782N, G676V, K165*, and E717*) were identified in three out of four patients [50]. However, no mutations in *CTNNB1* were identified in the same tumors. Axin2, like axin1, acts as a scaffold to help assemble the β-catenin destruction complex; both proteins show high similarity and are considered functionally equivalent. However, only axin2, but not axin1, is a transcriptional target of β-catenin–dependent Wnt signaling. Thus, axin2 protein levels could be a key negative feedback mechanism for the regulation of Wnt/β-catenin signaling in cells [51]. The frequency of mutations in the *AXIN2* gene in patients with CCA is also very low (0.7% and 1.1% in COSMIC and CBioPortal, respectively) (Table 1). The analysis by NGS of samples from 66 Chinese patients with CCA (44 iCCAs and 22 eCCAs) identified mutations in the *AXIN2* gene only in two of these patients (3%), both female, one of each type of tumor, and alterations were associated with tumor mutational burden [52]. Genetic alterations in *AXIN2* have been associated with poor prognosis in other early-stage solid tumors [53]. Other genes encoding Wnt/β-catenin pathway proteins less frequently mutated in CCA are *DVL2*, *DVL3*, *FZD10*, *WNT10B*, and *WNT8B* (in ~1% of cases each; for a review, see [35]).

### 2.4. Alterations in Wnt Ligands

The Wnt/β-catenin signaling pathway has multiple nodes of interaction with other signaling cascades and is regulated in a complex cellular environment. It has been described that the Wnt/β-catenin pathway is highly active in CCA because this tumor presents a high Wnt state, which is maintained by inflammatory macrophages in the surrounding stroma [54]. In this regard, it has been described that the level of Wnt ligands is higher in CCA tissue than in the adjacent non-tumor tissue. The analysis of 48 cases of liver fluke-derived iCCA showed that mRNA levels of Wnt3a, Wnt5a, and Wnt7b were higher in the tumor tissue than in the surrounding liver tissue. Immunohistochemical analysis of 38 iCCA cases detected Wnt3a protein in the cytoplasm of tumor cells in 92.1% of cases, Wnt5a in 76.3% of cases, and Wnt7b in all tumors [55]. Moreover, the cumulative survival analysis demonstrated that CCA patients with Wnt5a positive expression had a significantly shorter survival time than those without Wnt5a expression [55]. Another study in 37 patients with different types of CCA (9 iCCA and 28 pCCA) described higher levels of Wnt7b and Wnt10a ligands in tumor samples compared with matched non-tumor tissues [54]. Using an experimental model in rats with thioacetamide-induced CCA, it was demonstrated that the main source of the Wnt7b ligand was inflammatory macrophages [54]. In vitro evidence also supported that activated macrophages promote Wnt/β-catenin signaling in CCA cells [55].

### 2.5. Other Mechanisms Affecting Wnt/β-Catenin Pathway

In addition to high levels of Wnt factors, other mechanisms may increase the activity of the Wnt/β-catenin pathway in CCA. For example, it is well known that the transcription factor SOX17 is downregulated in CCA due to hypermethylation of its promoter [56]. SOX17 plays a role as a negative regulator of the Wnt/β-catenin pathway by interacting with β-catenin and hence inhibiting gene transcription [57,58]. SOX17 downregulation in CCA could help to maintain the high activity of this pathway. Furthermore, recent studies have shown that RNF43 is mutated in a subset of liver fluke-associated CCA in humans, resulting in a hyperactivated Wnt signaling cascade [59,60]. The reason is that RNF43 is an E3 ligase required for normal FZD receptor turnover after ligand binding, acting as an inhibitor of the Wnt/β-catenin pathway by reducing nuclear TCF expression and sequestering TCF4 at the nuclear membrane. Surprisingly, these mutated variants of RNF43 have not been detected in non-fluke-associated CCAs [60]. In vitro studies have shown that RNF43 expression levels are very low in different CCA cell lines, in which its experimentally induced expression attenuates β-catenin nuclear translocation, indicating that RNF43 is essential to prevent aberrant activation of the Wnt/β-catenin pathway [61]. In the same sense, CCA patients with tumors bearing inactivating mutations or downregulation of RNF43 have less favorable outcomes, supporting the consideration of *RNF43* as a tumor suppressor gene [60].

The expression levels of several microRNAs targeting elements of the Wnt/β-catenin pathway are altered in CCA compared with paired non-tumor tissue [62]. For example, increased levels of miRNA-26a have been observed in human CCA tissues compared with non-tumor biliary cells, which has been associated with the fact that this miRNA leads to a reduction in GSK-3β activity and thus the activation of the Wnt/β-catenin signaling pathway [63]. Similarly, miRNA-221 and miRNA-191, whose expression levels are increased in CCA tumors and CCA-derived cells, have been shown to activate β-catenin signaling [64,65].

## 3. β-Catenin Heterogeneity in CCA and Prognosis

**Figure 2:**
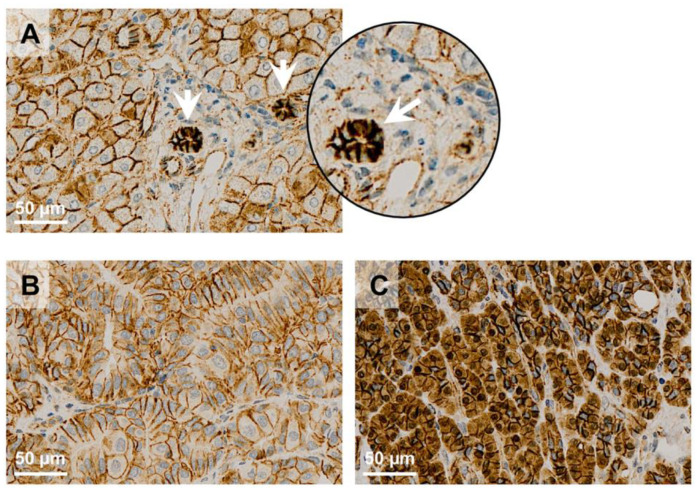
β-catenin immunohistochemistry showing basolateral membrane staining in normal adult human liver hepatocytes (**A**), with an evident greater intensity in bile duct cells ((**A**), arrows). Absence of β-catenin nuclear immunoreactivity in CCA cells due to Wnt/β-catenin signaling pathway inactivation (**B**). Strong nuclear and cytoplasmic β-catenin staining in CCA cells with Wnt/β-catenin signaling pathway activation (**C**). Anti-β-catenin antibody clone 17C2 (Leica Biosystems) was used [66].

The expression of β-catenin is higher in cholangiocytes than in hepatocytes. Due to the association of β-catenin with E-cadherin-based adherens junctions, in healthy liver, β-catenin is localized at the plasma membrane and, to a much lesser extent, also in the cytoplasm of both types of liver cells (Figure 2A). Compared with non-tumor liver cells, malignant cholangiocytes show a remarkable heterogeneity in the expression levels of this protein (from preserved to a very low expression) and also regarding its subcellular localization (plasma membrane, cytoplasm, and nuclei) (Figure 2B,C). The down-regulation of epithelial markers is a characteristic alteration of the epithelial–mesenchymal transition (EMT), but in the case of β-catenin, it also reflects the activation of this signaling pathway.

Several studies have investigated β-catenin expression and localization in CCA by immunohistochemistry (Table 2), since the increased staining of β-catenin in cytoplasm and nuclei by this technique is considered the best method to detect the activation of the Wnt/β-catenin pathway.

The reduction in the presence of β-catenin at the plasma membrane usually parallels a decrease in the expression levels of other intercellular junction markers, such as E-cadherin or α-catenin [67,69]. β-catenin nuclear expression is not observed in normal liver, but it has been detected in many (2–50%) CCAs (Table 2).

There is controversy regarding the existence of differences in the frequency of nuclear staining of β-catenin depending on the CCA type. One study reported higher nuclear levels of β-catenin in pCCA than in iCCA [74], whereas another study described higher levels in iCCA than in eCCA [75]. The small number of cases in some studies, the use of different criteria in staining analysis, and recent changes in the classification of CCAs do not allow us to reach robust conclusions on this point. Nevertheless, it is generally accepted that (i) aberrant nuclear expression is associated with CCA malignancy, (ii) nuclear translocation does not require genetic mutations [68], and (iii) the reduced expression of β-catenin at the plasma membrane and its overexpression in the nucleus are two factors associated with poor prognosis [70,73]. In addition, frequent nuclear localization of β-catenin has been observed in cancer cells forming the invasive front of CCA [72], as has also been found in other tumors [78].

In patients with cHCC-CCA, a relationship between reduced β-catenin membranous expression and tumor progression and metastasis, together with higher differentiation of HCC components, has been found [77]. In that cohort, the nuclear accumulation of β-catenin was observed in only two cases and only in HCC-related cells. Interestingly, in cases of cHCC-CCA with intrahepatic metastasis, the subcellular expression pattern of β-catenin in the secondary tumor cells was frequently found to be similar to those found in the primary lesions [77]. The low frequency of nuclear location compared to previously available data obtained both in HCC and CCA makes further studies in this subtype of tumors imperative to determine whether they have a particularly different behavior.

CCA-derived cell lines have been helpful in investigating the association of β-catenin activation and tumor cell aggressiveness and in further understanding the functioning of this signaling pathway. Higher protein expression levels of β-catenin were found in the metastasizing cell line OZ compared with the moderately invasive cell line HuCCT1, but β-catenin expression did not account for cell invasiveness in the OZ cell line [79]. The analysis of β-catenin expression in pCCA-(FRH0201) and iCCA-(HCCC-9810, RBE, and SSP-25) derived cell lines revealed different expression order, depending on whether this was based on the abundance of mRNA determined by RT-qPCR (FRH0201 ≈ HCCC-9810 > SSP-25 > RBE) or protein determined by Western blot (HCCC-9810 > FRH0201 > RBE > SSP-25). In all cases, β-catenin was detected in the plasma membrane, cytoplasm, and nuclei by immunofluorescence; the most intense signal was found in FRH0201 and HCCC-9810 cells [43]. RNAi targeting β-catenin or its ligand Wnt2 downregulated the expression of these two genes in FRH0201 cells, inhibited the activation of the Wnt pathway, and downregulated the mRNA expression levels of c-myc, promoting cell apoptosis, arresting the cell cycle at the G0/G1 phase, and inhibiting cell proliferation [43].

β-catenin has been linked to the development of a more malignant phenotype in initiated cholangiocytes, since exposing these cells to exosomes isolated from CCA cells resulted in the nuclear expression of β-catenin [80]. The tumor microenvironment has been shown to modulate tumor growth and metastasis. Co-culture of CCA cells QBC939 and Mz-ChA-1 with mesenchymal stem cells, or with their conditioned media, increased colony formation and invasion of tumor cells via Wnt/β-catenin [81]. It has been described that intramembranous proteolysis of epithelial cell adhesion molecule (EpCAM) releases an epithelial cell intracellular domain (EpICD) into the cytoplasm, which translocates to the nucleus and forms a complex with β-catenin. In surgical samples of eCCA, a positive correlation between the nuclear levels of EpICD and β-catenin was observed by immunohistochemistry [72]. In the CCA cell lines, the forced expression of EpICD stimulates cell growth and proliferation and also the expression levels of the active form of β-catenin and EpCAM target genes, such as c-myc and cyclin D1 [72].

## 4. Potential Therapies Targeting Wnt/β-Catenin Pathway in CCA

Considering the importance of the Wnt pathway in the tumorigenesis of several types of cancer, a great effort has been made to find compounds able to inhibit the activity of this pathway. In this sense, many patents in the area of Wnt modulation have been filed, and some small molecules or antibodies have been included in clinical trials to evaluate their usefulness as Wnt modulators (for a review see [82]). The most popular therapeutic approaches have consisted of molecules against Wnt ligands or against FZD receptors or compounds that inhibit Wnt ligand secretion or their interaction with their receptor. Other options include inhibition of the palmitoylation of Wnt ligands. Additional agents have been developed to promote β-catenin degradation in the cytoplasm and to inhibit its nuclear translocation [83]. Given the impact of the altered Wnt/β-catenin pathway on CCA, several studies have been designed to explore the usefulness of suppressing Wnt/β-catenin signaling in preclinical and clinical assays in CCA.

One strategy to reduce Wnt/β-catenin signaling is the inhibition of porcupine, a membrane-bound O-acyltransferase that catalyzes the acylation of Wnt proteins [35]. This post-translational modification is essential for the secretion and correct function of Wnt ligands. Accordingly, the porcupine has been suggested as a potential target to prevent the activation of the Wnt/β-catenin pathway by interfering with the functionality of the pathway activators. In this sense, some small molecules and monoclonal antibodies against porcupine are being tested in clinical trials. CGX1321, a small peptide that inhibits porcupine, is currently being tested in a phase I trial in patients with advanced solid tumors, including gastrointestinal tumors (NCT02675946). C-59 is another inhibitor of this enzyme that has shown its ability to inhibit Wnt signaling by reducing CCA proliferation in animal models [54]. In the same sense, the porcupine inhibitor LGK-974 has been demonstrated to reduce CCA cell proliferation in vitro in both mucin and mixed iCCA models [84]. It is also important to mention that this inhibitor has been tested in vivo [85] and is being tested in a phase I clinical trial for the treatment of Wnt-dependent malignancies, such as colorectal or pancreatic cancer (NCT01351103) [86].

Dickkopf-1 (DKK1) is upregulated in many types of cancer, and its inhibition has been associated with decreased tumor proliferation, migration, and invasion in preclinical trials [87]. DKK1 has been shown to regulate tumorigenicity and invasion in CCA through the β-catenin/matrix metalloproteinase-7 (MMP-7) pathway [87], in addition to promoting tumor immune evasion through the recruitment of immune suppressive macrophages, [88] and has therefore been proposed as a potential therapeutic target. A humanized IgG4 monoclonal antibody (DKN-01) has been developed to bind to DKK1 and block its activity. In two mouse models of iCCA, the inhibition of DKK1 with DKN-01 resulted in a reduced tumor burden [88]. This monoclonal antibody has been tested in a phase I trial in combination with gemcitabine and cisplatin in patients with advanced CCA (NCT02375880). Preliminary results have indicated that DKN-01 is well tolerated in this combination but did not appear to improve activity over the administration of gemcitabine/cisplatin alone [89]. Nonetheless, a study with a higher dose of DKN-01 in combination with a programmed cell death protein 1 (PD-1) inhibitor in biliary tract cancer is ongoing.

Another possibility for inhibiting this pathway is to block transcription of β-catenin target genes by preventing the nuclear interaction between β-catenin and its transcription factors TCF/LEF. Several known drugs and natural molecules have this effect, such as vitamin D and retinoic acid [90,91] or the small molecule ICG-001, which has shown effectivity inhibiting the pathway and reducing CCA proliferation in several animal models of iCCA [54]. An isomer of ICG-001 named PRI-724 is currently being tested in phase I clinical trials for advanced myeloid malignancies (NCT01606579), and the results of another phase I trial in which it was administered in combination with gemcitabine for metastatic pancreatic adenocarcinoma are being evaluated (NCT01764477). Although the effectiveness and safety of PRI-724 in the treatment of patients with CCA have not yet been demonstrated, this compound has been evaluated (phase 1/2a study) in patients with hepatitis C and B virus-induced liver cirrhosis. The results indicated that this compound is well tolerated in patients with liver cirrhosis [92].

Another compound with a similar mechanism of action is BC2059 (tegavivint), a Wnt/β-catenin pathway inhibitor that disrupts the binding of β-catenin to Transducin β-like protein 1 (TBL1), a key player in enhancing the canonical Wnt signaling pathway via direct binding to β-catenin and recruiting it to the promoter of Wnt target genes, thereby facilitating β-catenin destruction [93]. In vitro experiments using myeloma and osteosarcoma cell lines have shown that this compound decreases β-catenin nuclear levels and reduces the expression of Wnt target genes. BC2059 has been tested in a phase I clinical trial focused on patients with progressive desmoid tumors (NCT03459469), whose results have not yet been published.

In addition, the non-steroidal anti-inflammatory drugs sulindac and aspirin have proved to promote the degradation of β-catenin [94]. The effects on the Wnt/β-catenin pathway could partly justify the beneficial effects observed with vitamin D, aspirin, or sulindac in preclinical and/or clinical studies in the treatment of CCA and other cancers [95,96,97].

Different preclinical studies have shown that docosahexaenoic acid (DHA), an omega 3 polyunsaturated fatty acid, induces GSK3β dephosphorylation in human CCA cells [98]. This activation of GSK3β promotes Axin assembly with GSK3β, favoring the formation of the β-catenin destruction complex, which leads to β-catenin degradation [99]. Therefore, DHA treatment decreases β-catenin–mediated TCF/LEF reporter activity and inhibits the expression of c-Met, a β-catenin target gene involved in cholangiocarcinogenesis [99].

miRNAs have been identified that play a role in regulating the activity of the Wnt/β-catenin pathway. Specifically, a preclinical study showed that inhibition of miRNA-26a prevented β-catenin activation and transcription of its target genes and slowed the rate of tumor growth, an effect caused by this microRNA blocking the GSK3β mRNA [63]. Similarly, MIR22HG, a long non-coding RNA that is downregulated in CCA cell lines and tissues [100], decreases β-catenin mRNA and protein levels. In vitro experiments demonstrated that overexpression of MIR22HG decreased cell proliferation, migration, and invasion, reducing β-catenin levels and thus inducing downregulation of the target genes of this pathway, *MYC* and *CCND1* [100].

In vitro and in vivo assays have shown that restoration of SOX17, whose expression is markedly reduced in CCA compared to healthy cholangiocytes, decreases the activity of the Wnt/β-catenin pathway, reducing tumor proliferation and progression [56]. Experimentally forced SOX17 expression in CCA cells induced selective chemosensitization to 5-fluorouracil, mitoxantrone, and SN-38 by reducing their cellular efflux through multidrug resistance-associated protein 3 (MRP3) [101]. In the same way, inhibition of this pathway in CCA cell lines with β-catenin siRNA leads to the overcoming of multidrug resistance; this effect is mediated by a reduction in the expression of the multidrug resistance protein (MDR1, P-glycoprotein) [102]. Similarly, in vitro studies using iCCA cells treated with the inhibitors of Wnt/β-catenin pathway C-59 and ICG-001, demonstrated higher sensitivity to 5-FU via modulation of ABC pumps and other genes related with different mechanisms of chemoresistance [66]. In another study, the administration of β-escin (a compound that induces β-catenin degradation) in combination with some of the most commonly used antitumor drugs for treating CCA patients (5-fluorouracil, vincristine sulfate, and mitomycin C) enhanced the response of human CCA cells due in part to reduced MDR1 protein expression [103].

It is important to note that although it was a priori thought that blocking the Wnt pathway would have no side effects in normal cells because this pathway is only activated in tumor cells, some side effects have been described upon using different inhibitory strategies. For example, the inhibition of the Wnt/β-catenin pathway disrupts intestinal homeostasis and induces severe loss of crypt-villi structure in mouse intestine [104]. Similarly, following Wnt blockade, tissue homeostasis is also impaired in hair follicles, stomach, and hematopoietic bone marrow, where Wnt signaling is essential for the maintenance of stem cells and their niches [105,106] (for a review, see [107]).

Several therapeutic agents specifically targeting the Wnt/β-catenin pathway have entered clinical trials, but none have yet been approved. Some trials in which Wnt pathway inhibitory agents have been administered in combination with chemotherapeutic drugs have obtained better results than monotherapy, without an increase of adverse effects [108]. Since these depend on various factors such as the dose, administration time, treatment period, and the intrinsic characteristics of each patient, it is necessary to carefully design and evaluate new Wnt inhibition strategies to improve their specificity and, therefore, their efficacy, and at the same time avoid possible adverse effects on healthy cells. Moreover, it is also necessary to identify novel biomarkers that permit the selection of patients who may benefit from these therapies.

## 5. Conclusions and Perspectives

The poor prognosis of most cases of advanced CCA constitutes a severe problem in modern oncology. The difficulty of dealing with this limitation is augmented by the heterogeneity of CCA subtypes and the complexity of mechanisms involved in enhanced proliferation, avoidance of apoptosis activation, chemoresistance, invasiveness, and metastasis. Among the regulatory processes involved in developing these malignant characteristics, the Wnt/β-catenin pathway plays a pivotal role. Alteration of β-catenin expression and subcellular localization has been associated with worse outcomes in some CCA subtypes. This heterogeneity, which also affects cellular and in vivo models of CCA, must be taken into account for CCA investigation. Recommendations on the minimal criteria for preclinical models of CCA to provide a uniform approach have been recently published [109] to more accurately extrapolate basic laboratory research to the clinical situation, helping to develop novel diagnostic tools and therapeutic strategies for patients suffering from this heterogeneous disease.

## Figures and Tables

**Table 1:** Mutations in genes encoding components of the Wnt/β-catenin pathway in CCA.

	COSMIC		CBioPortal	
	Frequency (%)	Cases (n)	Frequency (%)	Cases (n)
***APC***	3.1	1513	3.7	1361
***AXIN1***	2.2	803	1.0	1127
***AXIN2***	0.7	689	1.1	806
***CSNK1A1***	2.7	861	-	-
***CTNNB1***	1.8	1693	1.5	1223
***GSK3β***	0.3	665	-	-

Data obtained from COSMIC database for bile duct specimens and from the CBioPortal database for single intraductal papillary intraductal bile duct neoplasms.

**Table 2:** Altered expression of β-catenin in CCA.

Technique	Number of Cases	Reduced Membrane Expression	Nuclear Location	Reference
IHC	47 CCA	58%	NA	[67]
IHC	71 iCCA	82%	15%	[68]
IHC	20 CCA	NA	10%	[69]
IHC	31 iCCA	58%	16%	[70]
IHC	24 iCCA	cytoplasmic or nucleus staining in 58% of cases		[42]
IHC	83 CCA	38.6%	NA	[71]
IHC	79 eCCA	NA	12.6%	[72]
IHC	140 iCCA	cytoplasmic or nucleus staining in 46% of cases		[73]
IHC	31 iCCA 129 pCCA	72% 17%	18% 30%	[74]
IHC	6 iCCA 12 eCCA 53 GBC	NA	50% 25% 49%	[75]
IHC	190 CCA	63%	2%	[76]
IHC	24 cHCC-CCA	33% CCA and 42% HCC components	8.3% HCC component	[77]

CCA, cholangiocarcinoma; cHCC-CCA, combined hepato-cholangiocarcinoma; eCCA, extrahepatic cholangiocarcinoma; GBC, gallbladder carcinoma; HCC, hepatocellular carcinoma; iCCA, intrahepatic cholangiocarcinoma; IHC, immunohistochemistry; NA, not available; pCCA, perihilar cholangiocarcinoma.

## References

[1] Banales J.M., Marin J.J.G., Lamarca A., Rodrigues P.M., Khan S.A., Roberts L.R., Cardinale V., Carpino G., Andersen J.B., Braconi C. (2020). Cholangiocarcinoma 2020: The next horizon in mechanisms and management. Nat. Rev. Gastroenterol. Hepatol..

[2] Izquierdo-Sanchez L., Lamarca A., La Casta A., Buettner S., Utpatel K., Klumpen H.J., Adeva J., Vogel A., Lleo A., Fabris L. (2022). Cholangiocarcinoma landscape in Europe: Diagnostic, prognostic and therapeutic insights from the ENSCCA Registry. J. Hepatol..

[3] Shroff R.T., Javle M.M., Xiao L., Kaseb A.O., Varadhachary G.R., Wolff R.A., Raghav K.P.S., Iwasaki M., Masci P., Ramanathan R.K. (2019). Gemcitabine, Cisplatin, and nab-Paclitaxel for the Treatment of Advanced Biliary Tract Cancers: A Phase 2 Clinical Trial. JAMA Oncol..

[4] Macias R.I.R., Cardinale V., Kendall T.J., Avila M.A., Guido M., Coulouarn C., Braconi C., Frampton A.E., Bridgewater J., Overi D. (2022). Clinical relevance of biomarkers in cholangiocarcinoma: Critical revision and future directions. Gut.

[5] Clements O., Eliahoo J., Kim J.U., Taylor-Robinson S.D., Khan S.A. (2020). Risk factors for intrahepatic and extrahepatic cholangiocarcinoma: A systematic review and meta-analysis. J. Hepatol..

[6] Kamsa-ard S., Kamsa-ard S., Luvira V., Suwanrungruang K., Vatanasapt P., Wiangnon S. (2018). Risk Factors for Cholangiocarcinoma in Thailand: A Systematic Review and Meta-Analysis. Asian Pac. J. Cancer Prev..

[7] Rizvi S., Gores G.J. (2014). Molecular pathogenesis of cholangiocarcinoma. Dig. Dis..

[8] Lozano E., Sanchez-Vicente L., Monte M.J., Herraez E., Briz O., Banales J.M., Marin J.J., Macias R.I. (2014). Cocarcinogenic effects of intrahepatic bile acid accumulation in cholangiocarcinoma development. Mol. Cancer Res..

[9] Kongpetch S., Jusakul A., Ong C.K., Lim W.K., Rozen S.G., Tan P., Teh B.T. (2015). Pathogenesis of cholangiocarcinoma: From genetics to signalling pathways. Best. Pract. Res. Clin. Gastroenterol..

[10] Sia D., Hoshida Y., Villanueva A., Roayaie S., Ferrer J., Tabak B., Peix J., Sole M., Tovar V., Alsinet C. (2013). Integrative molecular analysis of intrahepatic cholangiocarcinoma reveals 2 classes that have different outcomes. Gastroenterology.

[11] Andersen J.B., Thorgeirsson S.S. (2013). Genomic decoding of intrahepatic cholangiocarcinoma reveals therapeutic opportunities. Gastroenterology.

[12] Isomoto H., Kobayashi S., Werneburg N.W., Bronk S.F., Guicciardi M.E., Frank D.A., Gores G.J. (2005). Interleukin 6 upregulates myeloid cell leukemia-1 expression through a STAT3 pathway in cholangiocarcinoma cells. Hepatology.

[13] Sirica A.E. (2008). Role of ErbB family receptor tyrosine kinases in intrahepatic cholangiocarcinoma. World J. Gastroenterol..

[14] Brivio S., Cadamuro M., Strazzabosco M., Fabris L. (2017). Tumor reactive stroma in cholangiocarcinoma: The fuel behind cancer aggressiveness. World J. Hepatol..

[15] Niessen C.M., Gottardi C.J. (2008). Molecular components of the adherens junction. Biochim. Biophys. Acta.

[16] Stamos J.L., Weis W.I. (2013). The beta-catenin destruction complex. Cold Spring Harb. Perspect. Biol..

[17] Cadigan K.M., Waterman M.L. (2012). TCF/LEFs and Wnt signaling in the nucleus. Cold Spring Harb. Perspect. Biol..

[18] Kaidi A., Williams A.C., Paraskeva C. (2007). Interaction between beta-catenin and HIF-1 promotes cellular adaptation to hypoxia. Nat. Cell Biol..

[19] Essers M.A., de Vries-Smits L.M., Barker N., Polderman P.E., Burgering B.M., Korswagen H.C. (2005). Functional interaction between beta-catenin and FOXO in oxidative stress signaling. Science.

[20] Kormish J.D., Sinner D., Zorn A.M. (2010). Interactions between SOX factors and Wnt/beta-catenin signaling in development and disease. Dev. Dyn..

[21] MacDonald B.T., Tamai K., He X. (2009). Wnt/beta-catenin signaling: Components, mechanisms, and diseases. Dev. Cell.

[22] Adler P.N. (2002). Planar signaling and morphogenesis in Drosophila. Dev. Cell.

[23] Nusse R. (2012). Wnt signaling. Cold Spring Harb. Perspect. Biol..

[24] Yang Y., Mlodzik M. (2015). Wnt-Frizzled/planar cell polarity signaling: Cellular orientation by facing the wind (Wnt). Annu. Rev. Cell Dev. Biol..

[25] Masyuk T.V., Huang B.Q., Ward C.J., Masyuk A.I., Yuan D., Splinter P.L., Punyashthiti R., Ritman E.L., Torres V.E., Harris P.C. (2003). Defects in cholangiocyte fibrocystin expression and ciliary structure in the PCK rat. Gastroenterology.

[26] Alvaro D., Onori P., Alpini G., Franchitto A., Jefferson D.M., Torrice A., Cardinale V., Stefanelli F., Mancino M.G., Strazzabosco M. (2008). Morphological and functional features of hepatic cyst epithelium in autosomal dominant polycystic kidney disease. Am. J. Pathol..

[27] Chu A.S., Russo P.A., Wells R.G. (2012). Cholangiocyte cilia are abnormal in syndromic and non-syndromic biliary atresia. Mod. Pathol..

[28] Mansini A.P., Peixoto E., Thelen K.M., Gaspari C., Jin S., Gradilone S.A. (2018). The cholangiocyte primary cilium in health and disease. Biochim. Biophys. Acta Mol. Basis Dis..

[29] Karjoo S., Hand N.J., Loarca L., Russo P.A., Friedman J.R., Wells R.G. (2013). Extrahepatic cholangiocyte cilia are abnormal in biliary atresia. J. Pediatr. Gastroenterol. Nutr..

[30] Fabris L., Fiorotto R., Spirli C., Cadamuro M., Mariotti V., Perugorria M.J., Banales J.M., Strazzabosco M. (2019). Pathobiology of inherited biliary diseases: A roadmap to understand acquired liver diseases. Nat. Rev. Gastroenterol. Hepatol..

[31] Gradilone S.A., Radtke B.N., Bogert P.S., Huang B.Q., Gajdos G.B., LaRusso N.F. (2013). HDAC6 inhibition restores ciliary expression and decreases tumor growth. Cancer Res..

[32] Fabbri L., Bost F., Mazure N.M. (2019). Primary Cilium in Cancer Hallmarks. Int. J. Mol. Sci..

[33] Morgan D., Eley L., Sayer J., Strachan T., Yates L.M., Craighead A.S., Goodship J.A. (2002). Expression analyses and interaction with the anaphase promoting complex protein Apc2 suggest a role for inversin in primary cilia and involvement in the cell cycle. Hum. Mol. Genet..

[34] Simons M., Gloy J., Ganner A., Bullerkotte A., Bashkurov M., Kronig C., Schermer B., Benzing T., Cabello O.A., Jenny A. (2005). Inversin, the gene product mutated in nephronophthisis type II, functions as a molecular switch between Wnt signaling pathways. Nat. Genet..

[35] Perugorria M.J., Olaizola P., Labiano I., Esparza-Baquer A., Marzioni M., Marin J.J.G., Bujanda L., Banales J.M. (2019). Wnt-beta-catenin signalling in liver development, health and disease. Nat. Rev. Gastroenterol. Hepatol..

[36] Kim Y.D., Park C.H., Kim H.S., Choi S.K., Rew J.S., Kim D.Y., Koh Y.S., Jeung K.W., Lee K.H., Lee J.S. (2008). Genetic alterations of Wnt signaling pathway-associated genes in hepatocellular carcinoma. J. Gastroenterol. Hepatol..

[37] Segditsas S., Tomlinson I. (2006). Colorectal cancer and genetic alterations in the Wnt pathway. Oncogene.

[38] Polakis P. (2012). Wnt signaling in cancer. Cold Spring Harb. Perspect. Biol..

[39] Koch A., Denkhaus D., Albrecht S., Leuschner I., von Schweinitz D., Pietsch T. (1999). Childhood hepatoblastomas frequently carry a mutated degradation targeting box of the beta-catenin gene. Cancer Res..

[40] Cairo S., Armengol C., De Reyniès A., Wei Y., Thomas E., Renard C.A., Goga A., Balakrishnan A., Semeraro M., Gresh L. (2008). Hepatic stem-like phenotype and interplay of Wnt/beta-catenin and Myc signaling in aggressive childhood liver cancer. Cancer Cell.

[41] Eichenmuller M., Trippel F., Kreuder M., Beck A., Schwarzmayr T., Haberle B., Cairo S., Leuschner I., von Schweinitz D., Strom T.M. (2014). The genomic landscape of hepatoblastoma and their progenies with HCC-like features. J. Hepatol..

[42] Tokumoto N., Ikeda S., Ishizaki Y., Kurihara T., Ozaki S., Iseki M., Shimizu Y., Itamoto T., Arihiro K., Okajima M. (2005). Immunohistochemical and mutational analyses of Wnt signaling components and target genes in intrahepatic cholangiocarcinomas. Int. J. Oncol..

[43] Zhang K.S., Zhou Q., Wang Y.F., Liang L.J. (2013). Inhibition of Wnt signaling induces cell apoptosis and suppresses cell proliferation in cholangiocarcinoma cells. Oncol. Rep..

[44] Simbolo M., Fassan M., Ruzzenente A., Mafficini A., Wood L.D., Corbo V., Melisi D., Malleo G., Vicentini C., Malpeli G. (2014). Multigene mutational profiling of cholangiocarcinomas identifies actionable molecular subgroups. Oncotarget.

[45] Cong W.M., Bakker A., Swalsky P.A., Raja S., Woods J., Thomas S., Demetris A.J., Finkelstein S.D. (2001). Multiple genetic alterations involved in the tumorigenesis of human cholangiocarcinoma: A molecular genetic and clinicopathological study. J. Cancer Res. Clin. Oncol..

[46] Churi C.R., Shroff R., Wang Y., Rashid A., Kang H.C., Weatherly J., Zuo M., Zinner R., Hong D., Meric-Bernstam F. (2014). Mutation profiling in cholangiocarcinoma: Prognostic and therapeutic implications. PLoS ONE.

[47] Lee C.H., Wang H.E., Seo S.Y., Kim S.H., Kim I.H., Kim S.W., Lee S.T., Kim D.G., Han M.K., Lee S.O. (2016). Cancer related gene alterations can be detected with next-generation sequencing analysis of bile in diffusely infiltrating type cholangiocarcinoma. Exp. Mol. Pathol..

[48] Yang B., House M.G., Guo M., Herman J.G., Clark D.P. (2005). Promoter methylation profiles of tumor suppressor genes in intrahepatic and extrahepatic cholangiocarcinoma. Mod. Pathol..

[49] Capuozzo M., Santorsola M., Landi L., Granata V., Perri F., Celotto V., Gualillo O., Nasti G., Ottaiano A. (2022). Evolution of Treatment in Advanced Cholangiocarcinoma: Old and New towards Precision Oncology. Int. J. Mol. Sci..

[50] Mimaki S., Totsuka Y., Suzuki Y., Nakai C., Goto M., Kojima M., Arakawa H., Takemura S., Tanaka S., Marubashi S. (2016). Hypermutation and unique mutational signatures of occupational cholangiocarcinoma in printing workers exposed to haloalkanes. Carcinogenesis.

[51] Lee E., Salic A., Kruger R., Heinrich R., Kirschner M.W. (2003). The roles of APC and Axin derived from experimental and theoretical analysis of the Wnt pathway. PLoS Biol..

[52] Tian W., Hu W., Shi X., Liu P., Ma X., Zhao W., Qu L., Zhang S., Shi W., Liu A. (2020). Comprehensive genomic profile of cholangiocarcinomas in China. Oncol. Lett..

[53] Tseng R.C., Lin R.K., Wen C.K., Tseng C., Hsu H.S., Hsu W.H., Wang Y.C. (2008). Epigenetic silencing of AXIN2/betaTrCP and deregulation of p53-mediated control lead to wild-type beta-catenin nuclear accumulation in lung tumorigenesis. Oncogene.

[54] Boulter L., Guest R.V., Kendall T.J., Wilson D.H., Wojtacha D., Robson A.J., Ridgway R.A., Samuel K., Van Rooijen N., Barry S.T. (2015). WNT signaling drives cholangiocarcinoma growth and can be pharmacologically inhibited. J. Clin. Investig..

[55] Loilome W., Bungkanjana P., Techasen A., Namwat N., Yongvanit P., Puapairoj A., Khuntikeo N., Riggins G.J. (2014). Activated macrophages promote Wnt/beta-catenin signaling in cholangiocarcinoma cells. Tumour Biol..

[56] Merino-Azpitarte M., Lozano E., Perugorria M.J., Esparza-Baquer A., Erice O., Santos-Laso A., O’Rourke C.J., Andersen J.B., Jimenez-Aguero R., Lacasta A. (2017). SOX17 regulates cholangiocyte differentiation and acts as a tumor suppressor in cholangiocarcinoma. J. Hepatol..

[57] Sinner D., Rankin S., Lee M., Zorn A.M. (2004). Sox17 and beta-catenin cooperate to regulate the transcription of endodermal genes. Development.

[58] Sinner D., Kordich J.J., Spence J.R., Opoka R., Rankin S., Lin S.C., Jonatan D., Zorn A.M., Wells J.M. (2007). Sox17 and Sox4 differentially regulate beta-catenin/T-cell factor activity and proliferation of colon carcinoma cells. Mol. Cell. Biol..

[59] Chan-On W., Nairismagi M.L., Ong C.K., Lim W.K., Dima S., Pairojkul C., Lim K.H., McPherson J.R., Cutcutache I., Heng H.L. (2013). Exome sequencing identifies distinct mutational patterns in liver fluke-related and non-infection-related bile duct cancers. Nat. Genet..

[60] Ong C.K., Subimerb C., Pairojkul C., Wongkham S., Cutcutache I., Yu W., McPherson J.R., Allen G.E., Ng C.C., Wong B.H. (2012). Exome sequencing of liver fluke-associated cholangiocarcinoma. Nat. Genet..

[61] Pangestu N.S., Chueakwon P., Talabnin K., Khiaowichit J., Talabnin C. (2021). RNF43 overexpression attenuates the Wnt/beta-catenin signalling pathway to suppress tumour progression in cholangiocarcinoma. Oncol. Lett..

[62] Goeppert B., Ernst C., Baer C., Roessler S., Renner M., Mehrabi A., Hafezi M., Pathil A., Warth A., Stenzinger A. (2016). Cadherin-6 is a putative tumor suppressor and target of epigenetically dysregulated miR-429 in cholangiocarcinoma. Epigenetics.

[63] Zhang J., Han C., Wu T. (2012). MicroRNA-26a promotes cholangiocarcinoma growth by activating beta-catenin. Gastroenterology.

[64] Li J., Yao L., Li G., Ma D., Sun C., Gao S., Zhang P., Gao F. (2015). miR-221 Promotes Epithelial-Mesenchymal Transition through Targeting PTEN and Forms a Positive Feedback Loop with beta-catenin/c-Jun Signaling Pathway in Extra-Hepatic Cholangiocarcinoma. PLoS ONE.

[65] Kang P.C., Leng K.M., Liu Y.P., Liu Y., Xu Y., Qin W., Gao J.J., Wang Z.D., Tai S., Zhong X.Y. (2018). miR-191 Inhibition Induces Apoptosis through Reactivating Secreted Frizzled-Related Protein-1 in Cholangiocarcinoma. Cell. Physiol. Biochem..

[66] Lozano E., Boulter L., Sanchón-Sánchez P., Briz O., Macias R.I.R., Marin J.J.G. (2023). Modulación de la ruta Wnt-ß-catenina como potencial estrategia quimiosensibilizante en el colangiocarcinoma. Gastroenterol. Hepatol..

[67] Ashida K., Terada T., Kitamura Y., Kaibara N. (1998). Expression of E-cadherin, alpha-catenin, beta-catenin, and CD44 (standard and variant isoforms) in human cholangiocarcinoma: An immunohistochemical study. Hepatology.

[68] Sugimachi K., Taguchi K., Aishima S., Tanaka S., Shimada M., Kajiyama K., Sugimachi K., Tsuneyoshi M. (2001). Altered expression of beta-catenin without genetic mutation in intrahepatic cholangiocarcinoma. Mod. Pathol..

[69] Yun K.J., Han W.C., Choi S.C., Kim T.H. (2002). Immunohistochemical Study of beta-catenin Expression between Hepatocellular Carcinoma and Cholangiocarcinoma. Cancer Res. Treat..

[70] Settakorn J., Kaewpila N., Burns G.F., Leong A.S. (2005). FAT, E-cadherin, beta catenin, HER 2/neu, Ki67 immuno-expression, and histological grade in intrahepatic cholangiocarcinoma. J. Clin. Pathol..

[71] Gu M.J., Choi J.H. (2012). Clinicopathological significance of E-cadherin, beta-catenin and epidermal growth factor receptor expression in intrahepatic cholangiocarcinoma. Hepatogastroenterology.

[72] Jachin S., Bae J.S., Sung J.J., Park H.S., Jang K.Y., Chung M.J., Kim D.G., Moon W.S. (2014). The role of nuclear EpICD in extrahepatic cholangiocarcinoma: Association with beta-catenin. Int. J. Oncol..

[73] Huang X.Y., Zhang C., Cai J.B., Shi G.M., Ke A.W., Dong Z.R., Zhang P.F., Fan J., Peng B.G., Zhou J. (2014). Comprehensive multiple molecular profile of epithelial mesenchymal transition in intrahepatic cholangiocarcinoma patients. PLoS ONE.

[74] Chen W., Liang J., Huang L., Cai J., Lei Y., Lai J., Liang L., Zhang K. (2016). Characterizing the activation of the Wnt signaling pathway in hilar cholangiocarcinoma using a tissue microarray approach. Eur. J. Histochem..

[75] Papadopoulou K., Murray S., Manousou K., Tikas I., Dervenis C., Sgouros J., Rontogianni D., Lakis S., Bobos M., Poulios C. (2018). Genotyping and mRNA profiling reveal actionable molecular targets in biliary tract cancers. Am. J. Cancer Res..

[76] Padthaisong S., Thanee M., Namwat N., Phetcharaburanin J., Klanrit P., Khuntikeo N., Titapun A., Loilome W. (2020). A panel of protein kinase high expression is associated with postoperative recurrence in cholangiocarcinoma. BMC Cancer.

[77] Asayama Y., Taguchi K., Aishima Si S., Nishi H., Masuda K., Tsuneyoshi M. (2002). The mode of tumour progression in combined hepatocellular carcinoma and cholangiocarcinoma: An immunohistochemical analysis of E-cadherin, alpha-catenin and beta-catenin. Liver.

[78] Paul S., Dey A. (2008). Wnt signaling and cancer development: Therapeutic implication. Neoplasma.

[79] Abuetabh Y., Persad S., Nagamori S., Huggins J., Al-Bahrani R., Sergi C. (2011). Expression of E-cadherin and beta-catenin in two cholangiocarcinoma cell lines (OZ and HuCCT1) with different degree of invasiveness of the primary tumor. Ann. Clin. Lab. Sci..

[80] Dutta S., Reamtong O., Panvongsa W., Kitdumrongthum S., Janpipatkul K., Sangvanich P., Piyachaturawat P., Chairoungdua A. (2015). Proteomics profiling of cholangiocarcinoma exosomes: A potential role of oncogenic protein transferring in cancer progression. Biochim. Biophys. Acta.

[81] Wang W., Zhong W., Yuan J., Yan C., Hu S., Tong Y., Mao Y., Hu T., Zhang B., Song G. (2015). Involvement of Wnt/beta-catenin signaling in the mesenchymal stem cells promote metastatic growth and chemoresistance of cholangiocarcinoma. Oncotarget.

[82] Goswami V.G., Patel B.D. (2021). Recent updates on Wnt signaling modulators: A patent review (2014–2020). Expert. Opin. Ther. Pat..

[83] Pez F., Lopez A., Kim M., Wands J.R., Caron de Fromentel C., Merle P. (2013). Wnt signaling and hepatocarcinogenesis: Molecular targets for the development of innovative anticancer drugs. J. Hepatol..

[84] Fraveto A., Cardinale V., Bragazzi M.C., Giuliante F., De Rose A.M., Grazi G.L., Napoletano C., Semeraro R., Lustri A.M., Costantini D. (2015). Sensitivity of Human Intrahepatic Cholangiocarcinoma Subtypes to Chemotherapeutics and Molecular Targeted Agents: A Study on Primary Cell Cultures. PLoS ONE.

[85] Liu J., Pan S., Hsieh M.H., Ng N., Sun F., Wang T., Kasibhatla S., Schuller A.G., Li A.G., Cheng D. (2013). Targeting Wnt-driven cancer through the inhibition of Porcupine by LGK974. Proc. Natl. Acad. Sci. USA.

[86] Du F.Y., Zhou Q.F., Sun W.J., Chen G.L. (2019). Targeting cancer stem cells in drug discovery: Current state and future perspectives. World J. Stem Cells.

[87] Shi X.D., Yu X.H., Wu W.R., Xu X.L., Wang J.Y., Xu L.B., Zhang R., Liu C. (2016). Dickkopf-1 expression is associated with tumorigenity and lymphatic metastasis in human hilar cholangiocarcinoma. Oncotarget.

[88] Jarman E.J., Horcas-Lopez M., Waddell S.H., MacMaster S., Gournopanos K., Soong D.Y.H., Musialik K.I., Tsokkou P., Ng M.E., Cambridge W.A. (2023). DKK1 drives immune suppressive phenotypes in intrahepatic cholangiocarcinoma and can be targeted with anti-DKK1 therapeutic DKN-01. Liver Int..

[89] Goyal L., Sirard C., Schrag M., Kagey M.H., Eads J.R., Stein S., El-Khoueiry A.B., Manji G.A., Abrams T.A., Khorana A.A. (2020). Phase I and Biomarker Study of the Wnt Pathway Modulator DKN-01 in Combination with Gemcitabine/Cisplatin in Advanced Biliary Tract Cancer. Clin. Cancer Res..

[90] Palmer H.G., Gonzalez-Sancho J.M., Espada J., Berciano M.T., Puig I., Baulida J., Quintanilla M., Cano A., de Herreros A.G., Lafarga M. (2001). Vitamin D(3) promotes the differentiation of colon carcinoma cells by the induction of E-cadherin and the inhibition of beta-catenin signaling. J. Cell Biol..

[91] Osei-Sarfo K., Gudas L.J. (2014). Retinoic acid suppresses the canonical Wnt signaling pathway in embryonic stem cells and activates the noncanonical Wnt signaling pathway. Stem Cells.

[92] Kimura K., Kanto T., Shimoda S., Harada K., Kimura M., Nishikawa K., Imamura J., Ogawa E., Saio M., Ikura Y. (2022). Safety, tolerability, and anti-fibrotic efficacy of the CBP/beta-catenin inhibitor PRI-724 in patients with hepatitis C and B virus-induced liver cirrhosis: An investigator-initiated, open-label, non-randomised, multicentre, phase 1/2a study. EBioMedicine.

[93] Fiskus W., Sharma S., Saha S., Shah B., Devaraj S.G., Sun B., Horrigan S., Leveque C., Zu Y., Iyer S. (2015). Pre-clinical efficacy of combined therapy with novel beta-catenin antagonist BC2059 and histone deacetylase inhibitor against AML cells. Leukemia.

[94] Sebio A., Kahn M., Lenz H.J. (2014). The potential of targeting Wnt/beta-catenin in colon cancer. Expert. Opin. Ther. Targets.

[95] Chiang K.C., Yeh C.N., Lin K.J., Su L.J., Yen T.C., Pang J.H., Kittaka A., Sun C.C., Chen M.F., Jan Y.Y. (2014). Chemopreventive and chemotherapeutic effect of dietary supplementation of vitamin D on cholangiocarcinoma in a Chemical-Induced animal model. Oncotarget.

[96] Choi J., Ghoz H.M., Peeraphatdit T., Baichoo E., Addissie B.D., Harmsen W.S., Therneau T.M., Olson J.E., Chaiteerakij R., Roberts L.R. (2016). Aspirin use and the risk of cholangiocarcinoma. Hepatology.

[97] Wentz S.C., Yip-Schneider M.T., Gage E.A., Saxena R., Badve S., Schmidt C.M. (2009). Sulindac prevents carcinogen-induced intrahepatic cholangiocarcinoma formation in vivo. J. Surg. Res..

[98] Zhang G.F., Qiu L., Yang S.L., Wu J.C., Liu T.J. (2020). Wnt/beta-catenin signaling as an emerging potential key pharmacological target in cholangiocarcinoma. Biosci. Rep..

[99] Lim K., Han C., Xu L., Isse K., Demetris A.J., Wu T. (2008). Cyclooxygenase-2-derived prostaglandin E2 activates beta-catenin in human cholangiocarcinoma cells: Evidence for inhibition of these signaling pathways by omega 3 polyunsaturated fatty acids. Cancer Res..

[100] Hu X., Tan Z., Yang Y., Yang P. (2019). Long non-coding RNA MIR22HG inhibits cell proliferation and migration in cholangiocarcinoma by negatively regulating the Wnt/beta-catenin signaling pathway. J. Gene Med..

[101] Lozano E., Asensio M., Perez-Silva L., Banales J.M., Briz O., Marin J.J.G. (2020). MRP3-Mediated Chemoresistance in Cholangiocarcinoma: Target for Chemosensitization Through Restoring SOX17 Expression. Hepatology.

[102] Shen D.Y., Zhang W., Zeng X., Liu C.Q. (2013). Inhibition of Wnt/beta-catenin signaling downregulates P-glycoprotein and reverses multi-drug resistance of cholangiocarcinoma. Cancer Sci..

[103] Huang G.L., Shen D.Y., Cai C.F., Zhang Q.Y., Ren H.Y., Chen Q.X. (2015). beta-escin reverses multidrug resistance through inhibition of the GSK3beta/beta-catenin pathway in cholangiocarcinoma. World J. Gastroenterol..

[104] Fevr T., Robine S., Louvard D., Huelsken J. (2007). Wnt/beta-catenin is essential for intestinal homeostasis and maintenance of intestinal stem cells. Mol. Cell. Biol..

[105] Duncan A.W., Rattis F.M., DiMascio L.N., Congdon K.L., Pazianos G., Zhao C., Yoon K., Cook J.M., Willert K., Gaiano N. (2005). Integration of Notch and Wnt signaling in hematopoietic stem cell maintenance. Nat. Immunol..

[106] Schepers A., Clevers H. (2012). Wnt signaling, stem cells, and cancer of the gastrointestinal tract. Cold Spring Harb. Perspect. Biol..

[107] Kim M.J., Huang Y., Park J.I. (2020). Targeting Wnt Signaling for Gastrointestinal Cancer Therapy: Present and Evolving Views. Cancers.

[108] Kahn M. (2014). Can we safely target the WNT pathway?. Nat. Rev. Drug. Discov..

[109] Calvisi D.F., Boulter L., Vaquero J., Saborowski A., Fabris L., Rodrigues P.M., Coulouarn C., Castro R.E., Segatto O., Raggi C. (2023). Criteria for preclinical models of cholangiocarcinoma: Scientific and medical relevance. Nat. Rev. Gastroenterol. Hepatol..

